# Traumatic brain injury and VA service use among Native Hawaiian and Pacific Islander Veterans experiencing homelessness

**DOI:** 10.3389/fpubh.2025.1639888

**Published:** 2025-09-19

**Authors:** Ryan Holliday, Lindsey Monteith, Shawn Liu, Sara Lum, Marissa Sia, Gayle Iwamasa, Darrin Aase, Alexandra Smith, Rishi Deka, Shiloh Jordan, Christine Kindler, Jack Tsai, Lisa A. Brenner

**Affiliations:** ^1^VA Pacific Islands Health Care System, Honolulu, HI, United States; ^2^VA Rocky Mountain Mental Illness Research, Education and Clinical Center for Suicide Prevention, Aurora, CO, United States; ^3^Departments of Psychiatry and Physical Medicine & Rehabilitation, University of Colorado Anschutz Medical Campus, Aurora, CO, United States; ^4^VA Homeless Programs Office, Washington, DC, United States; ^5^VA Office of Mental Health, Washington, DC, United States; ^6^VA National Center on Homelessness Among Veterans, Washington, DC, United States; ^7^School of Public Health, University of Texas Health Science Center, San Antonio, TX, United States; ^8^Department of Psychiatry, Yale University School of Medicine, New Haven, CT, United States; ^9^VA Eastern Colorado Health Care System, Aurora, CO, United States

**Keywords:** traumatic brain injury, homelessness, Veteran, Native Hawaiian and Pacific Islander, Veterans Affairs

## Abstract

**Introduction:**

United States Veterans experiencing homelessness often have myriad health concerns that can impact their functioning. Native Hawaiian or Pacific Islander (NHPI) homeless Veterans are up to 82% more likely to have a traumatic brain injury (TBI) diagnosis than non-NHPI homeless Veterans, which can further impact these Veterans’ psychosocial functioning. The Department of Veterans Affairs (VA) offers services to address the health and social needs of these Veterans.

**Methods:**

We examined VA electronic medical record data from 12,205 NHPI Veterans identified as homeless from 1/2005–7/2024. We calculated VA service use (i.e., homeless, justice, emergency, primary care, rehabilitative, mental health, and all other VA service settings) among NHPI homeless Veterans using Veteran electronic medical record data.

**Results:**

After accounting for sex, age, and VA service-connected disability, NHPI homeless Veterans with a documented TBI diagnosis were significantly more likely to access and use VA services across service settings.

**Discussion:**

NHPI homeless Veterans with TBI histories likely access various types of VA services; as such, wraparound care (e.g., Homeless Patient Aligned Care Teams; case management) are critical for managing care needs. Future research should examine factors that facilitate engagement in and benefit from VA health and social services among these Veterans.

## 1 Introduction

In the latest point-in-time count, it was estimated that 32,882 Veterans were experiencing homelessness (1). Toward this end, addressing the healthcare needs of homeless Veterans remains a top priority within the Department of Veterans Affairs [VA; (2, 3)]. To adequately meet the needs of Veterans experiencing homelessness, it is paramount to connect these Veterans to the myriad services the VA offers. In doing so, the complex health (e.g., cardiovascular conditions, mental health, traumatic brain injury [TBI]) and social (e.g., housing and employment assistance, navigation of the criminal legal system) needs of these Veterans can be better coordinated and addressed.

Nonetheless, it may be particularly challenging to reach specific subsets of the homeless Veteran population. There has been little research on Asian American Veterans and even less research on Native Hawaiian or Pacific Islander Veterans (NHPI) Veterans, despite the need for such research and various calls to disaggregate data from Asian American and NHPI populations (4, 5). Importantly, homeless Veterans who identify as Native Hawaiian or Pacific Islander (NHPI)^1^ are substantially less likely to use VA services, compared to non-NHPI and White Veterans (6). The research base examining factors that create barriers to service use among NHPI Veterans experiencing homelessness is nascent (7); however, several factors may impact accessing VA care, such as distrust of the VA system, desire for traditional healing approaches, mental health stigma, and shame (6–9). Further, a substantial portion of NHPI Veterans reside within the U.S. Pacific Islands (10), a region that is highly rural and geographically complex (e.g., distance between islands; transportation often requiring airplane travel).

Moreover, an unexpected finding previously reported by Holliday et al. (6) was that NHPI Veterans experiencing homelessness were also at substantially greater risk for having TBI documented in their VA medical record when compared to non-NHPI homeless Veterans. Specifically, after accounting for age, comorbid psychiatric diagnosis, and sex, NHPI homeless Veterans were nearly 82% more likely to have a TBI diagnosis relative to non-NHPI homeless Veterans.

TBI is an unfortunately common experience among those who have served. Although rates vary based on method of assessment, it is estimated that up to 67% of Veterans will experience at least one TBI in their lifetime (11) resulting in nearly 185,000 Veterans with a TBI history accessing VA care (12). TBI can impact numerous functional domains integral to engaging in VA services. For example, individuals with histories of TBI, particularly those moderate or severe in nature, can struggle with attention and memory, influencing the ability to remember appointments (13). Further, TBI-related difficulties with processing speed and distress tolerance can interfere with tracking complex health discussions and emotionally-valent treatment (e.g., trauma-focused psychotherapy; (13)), which can result in treatment non-adherence or attrition.

Nonetheless, despite the potential impact of neurocognitive dysfunction, some studies note that TBI history may result in increased VA service use (14), and that these Veterans’ clinical complexity may drive increased rates of screening, referral, and accessing care (i.e., attending and engaging in VA clinical encounters). However, prior studies have largely focused on overall VA as well as mental health and TBI-related (e.g., rehabilitation) service use (14). Moreover, such research has less frequently occurred with Veterans experiencing homelessness, who may access some VA services (e.g., emergency services) at a greater propensity than Veterans not experiencing housing instability (15).

No studies to date have focused on VA service use among NHPI homeless Veterans with histories of TBI. This gap in knowledge is problematic given the clinical complexity of this population and the contextual factors which may exacerbate these Veterans’ ability to access and engage in VA services (e.g., rurality). Building upon prior work, we examined the frequency of VA service use among homeless NHPI Veterans with histories of TBI across several VA settings, including homeless, justice, emergency, primary care, rehabilitative (e.g., spinal cord injury; TBI clinic), and mental health services. We hypothesized that TBI may be related to increased mental health and rehabilitative service use; however, we were unclear about the extent to which TBI would be associated with use of other VA services (e.g., in homeless or justice programs).

## 2 Materials and methods

We extracted data from the VA Corporate Data Warehouse (CDW) for Veterans identified as being homeless from 1/1/2005 to 7/31/2024. Identification of homelessness was based on a positive screen on the VA Homeless Screener,^2^ use of VA Homeless Programing based on stop codes, and/or International Classification of Diseases (ICD)-codes. For these Veterans, VA service use was identified as a Veteran attending a clinical encounter in the following VA service settings (i.e., homeless, justice, emergency, primary care, rehabilitative, mental health, and all other VA service settings). TBI diagnosis (based on ICD-codes; see (16)), race (i.e., NHPI identity), and additional sociodemographic data (i.e., age, sex) were also obtained through CDW. The total sample consisted of 12,205 NHPI Veterans experiencing homelessness.

We conducted a series of linear regressions to examine the association between TBI and each type of VA service use among NHPI Veterans, both unadjusted and adjusted for covariates (age, sex, and service connection). Given the nature of VA service use, which was skewed, both categorical (tertile, quartile) and transformed (logarithmic, square root) approaches were considered. After examining all approaches, a logarithmic transformation was found to adequately address skewedness and kurtosis and to be the most appropriate for modeling. All procedures were approved by the local Institutional Review Board and analyses were conducted with SPSS 29.0 on the VA network behind the firewall.

## 3 Results

Table 1 presents the sample characteristics of NHPI Veterans who experienced homelessness from 2005 to 2024. Service use varied across settings, with some settings having notably high rates of use. For example, the overwhelming majority of NHPI Veterans experiencing homelessness had at least one appointment in a VA mental health (99.3%; *n* = 12,125), primary care (94.6%; *n* = 11,551), or homeless (92.2%; 11,343) service setting. 15.2% (*n* = 1,860) of Veterans in this cohort had a TBI diagnosis.

**Table 1:** Sample characteristics.

Variable	*n*	%
Sex^a^		
Male	9,625	88.0
Female	1,307	12.0
Age^a^		
<34	3,392	31.0
35–54	5,445	49.8
>55	2,095	19.2
TBI diagnosis	1,860	15.2
VA service-connected disability	1,088	8.9
History of VA homeless program use	11,343	92.9
History of VA justice program use	1,807	14.8
History of VA emergency service use	3,508	28.7
History of VA primary care use	11,551	94.6
History of VA rehabilitative service use	5,775	47.3
History of VA mental health use	12,125	99.3
History of using any other VA services	9,517	78.0

a *n* = 1,273 data missing for sex and age.

NHPI, Native Hawaiian or Pacific Islander; TBI, traumatic brain injury.

In both unadjusted and adjusted models, NHPI Veterans experiencing homelessness with a TBI had greater use of VA services across all VA service settings when compared to those without a history of TBI (*p* < 0.01; Table 2). Among specific service settings, the association between TBI and service use appeared most robust among VA rehabilitative (adjusted *β* = 0.19), mental health (adjusted β = 0.18), all other VA (adjusted β = 0.16), and justice (adjusted β = 0.13) programing.

**Table 2:** Role of TBI diagnosis on VA service use among NHPI homeless Veterans.

VA Service setting	Unadjusted	Adjusted
	β	β
Homeless programs	0.03**	0.03**
Justice programs	0.15***	0.13***
Emergency services	0.06**	0.07***
Primary care	0.06***	0.12***
Rehabilitative services	0.17***	0.19***
Mental health	0.19***	0.18***
All other VA services	0.12***	0.16***

*p < 0.05 **p < 0.01 ***p < 0.001. All unadjusted and adjusted models were significant. VA service use logarithmically transformed for all service settings. Adjusted models covaried for sex, age, and VA service connection. NHPI, Native Hawaiian or Pacific Islander; TBI, traumatic brain injury.

## 4 Discussion

Among NHPI Veterans experiencing homelessness, those with a diagnosis of TBI were more likely to use VA services, including mental health and rehabilitative services, when compared to those without a TBI diagnosis. Building upon prior work, this finding also extended to other VA service settings commonly accessed by homeless Veterans, such as primary care, homeless and justice programs, and emergency services (17, 18).

Indeed, some prior research has found that Veterans with histories of TBI may have higher rates of VA service utilization (14). This may suggest that those experiencing homelessness and TBI-related neurocognitive impact (e.g., memory, executive functioning) may be more clinically complex, and therefore, require increased use of VA services (19). To this end, VA screens for TBI and has specialty services specifically tailored for Veterans with TBI histories. As such, greater service use among Veterans with TBI histories may be indicative of VA continuing to lead the field in conceptualizing and intervening upon TBI and associated sequelae (e.g., mental health conditions; unemployment). Indeed, VA has services designed to identify the complex needs of homeless Veterans and connect them to health and social services (e.g., case management; homeless patient aligned care teams). Further research is needed to identify specific factors related to accessing various types of VA care and how providers can best facilitate and tailor care to meet the needs of clinically complex homeless NHPI Veterans with TBI. For example, qualitative methods may prove particularly beneficial in informing facilitators, barriers, and provider experiences tailoring care for these Veterans.

These findings also support the need to further understand how services may need to be tailored for homeless Veterans with TBI histories, especially surrounding services to address social factors (e.g., unemployment). In such instances, psychoeducation, for the patient and associated entities (e.g., employer), and TBI-related care and accommodations may be warranted; nonetheless, we have limited understanding of care provided for these Veterans, particularly those who identify as NHPI. Further, it is likely that many of these Veterans accessed case management, which has the potential to facilitate access to VA services. Nonetheless, case management needs, including those found to be the most beneficial to the Veteran, remain understudied in this population. Additional approaches could be considered as well, including patient and provider resources on common TBI sequelae, as well as evidence-based treatments. Such an approach could facilitate functional recovery, as well as consideration of accommodations and tailoring to service delivery (e.g., increasing reminders, printing instructions) and activities of daily living (e.g., longer test taking time). Despite this, it is important to note that several potential confounding variables were not accounted for (e.g., posttraumatic stress disorder; comorbid serious mental illness), suggesting important next steps for understanding the intersection of TBI, homelessness, and multimorbidity among NHPI Veterans.

Importantly, NHPI Veterans experiencing homelessness appeared to have relatively high service use in several different VA settings, such as primary care and mental health. A potential explanation for this finding is that these settings often conduct initial biopsychosocial intakes and screening, during which homelessness and TBI history could be identified. These data are often then used, in turn, to inform referral to more specialty care (e.g., rehabilitation services). Given this, further research is needed to better understand what care NHPI homeless Veterans are accessing in these settings and if they may serve as critical intercepts for targeting delivery of TBI-related care for homeless NHPI Veterans. Further, in examining such health care workflows, systems of care can better understand how Veterans who identify as NHPI engage in care (e.g., accessing emergency or triage care to manage depression versus engaging in a course evidence-based treatment such as pharmaco- or psychotherapy) to inform methods of augmenting service delivery.

Given the extent to which NHPI Veterans experiencing homelessness also had a history of TBI, consideration of the intersection of health and culture is also critical. Health care within the U. S. has largely taken a biomedical approach, which may impact Veterans’ ability to discuss and approach their functional recovery in a holistic, patient-centered manner (e.g., barriers to communication, stigma, cultural mismatch with providers). As such, providers should consider local tenets in their biopsychosocial conceptualization. One example may be the inclusion of local nomenclature in the assessment and treatment process, as well as integrating NHPI concepts of health. The Hawaiian value of *Lōkahi*, or harmony, is representative of the balance between the physical, spiritual, and environmental (20). Harmony among these components is essential for holistic health (21). Pulotu-Endemann’s (22) *Fonofale* model of health represents a similar balance, with the model incorporating the Samoan *fale* or house, as a way to visualize essential components of health. For example, in this model, the family is represented by the foundation/floor; physical, spiritual, mental, and other identity factors are represented by the posts of the house; and culture is represented by the roof. All pieces of the *fale* are encircled by context, time, and the environment (22). These responsive practices not only respect the holistic health perspectives of NHPI Veterans but may also improve treatment adherence and outcomes by aligning care with their values and traditions (23, 24). Toward this end, integration of such holistic approaches may prove pragmatic for meeting the needs of NHPI Veterans experiencing homelessness and TBI. Potential methods could include dissemination of provider trainings on tailoring care as well as further evaluation of how best to integrate holistic care into existing evidence-based practices.

Several limitations are important to note. More nuanced information regarding Veterans’ experiences of homelessness, such as chronicity and number of episodes, were not included in our data. Similarly, these data were not cross-referenced with other administrative datasets, such as the VA’s Homeless Operations Management Evaluation System (25). NHPI identity was also based on electronic health records, and therefore, more nuanced examination of specific Pacific Islander ethnic groups (e.g., Chamorro, Samoan) could not be undertaken. In addition, given the timeframe and transient nature of this population, we could not include region as a covariate, limiting the ability to understand if these findings differ based on region (e.g., such as across the Pacific Islands). TBI was also categorized as a unidimensional variable, and there are various other clinically relevant variables that were not accounted for. The choice of treating TBI dichotomously was made given the number of providers diagnosing TBI without severity indicators (16), suggesting a need to further evaluate if findings are sustained across mild, moderate, and severe TBI designations. Although data reflected specific VA service settings, the types of care received in these settings, including completion of treatments and engagement, could not be examined. In addition, we did not account for other service use-related characteristics, including Veterans accessing multiple different types of VA services (e.g., concurrent primary and mental health care use). Finally, as data were cross-sectional in nature, longitudinal evaluation is requisite to determine if TBI is causally driving increased VA service use.

This project found that NHPI Veterans experiencing homelessness who also had a history of TBI were more likely than those without a documented TBI diagnosis to use every type of VA services examined. Further consideration of TBI-related education, resources, and augmentations to care for NHPI Veterans experiencing homelessness may be an important consideration. Integration of such factors should consider both evidence-based practice and cultural components that may enhance delivery of care to this population.

## Data Availability

The raw data supporting the conclusions of this article will be made available by the authors, without undue reservation, based on current local regulatory guidelines and statutes.

## Footnotes

^1^Many Veterans of NHPI descent may not identify with the term homeless given connections to the land in which they live providing a sense of being at home. Instead, terms such as “unhoused,” “houseless,” and “unsheltered” may more accurately describe this subset of this population of Veterans. However, as “homeless” is a standard VA definition (i.e., individuals without a full-time or adequate nighttime residence, living in a shelter or temporary living arrangement, or in a place not meant for human habitation), we will utilize this term throughout the remainder of the paper.

^2^See Montgomery et al. (26) for additional information regarding the VA Homeless Screener.
